# The role of demographic characteristics in US medical students’ professional well-being and medical school experiences: An intersectional approach

**DOI:** 10.1371/journal.pone.0338906

**Published:** 2025-12-16

**Authors:** Yuchen Liu, Aleeza Amin, Patricia Frazier

**Affiliations:** Department of Psychology, University of Minnesota-Twin Cities, Minneapolis, Minnesota, United States of America; Health Researcher, SPAIN

## Abstract

**Introduction:**

Previous findings have been mixed about the role of demographic characteristics in medical students’ well-being and school experiences when those characteristics were examined in isolation. The aim of this study was to investigate the roles of gender, race and ethnicity, and sexual orientation in medical students’ professional well-being and medical school experiences using an intersectional approach.

**Method:**

We analyzed data from the 2019–2022 Association of American Medical Colleges Graduation Questionnaire (*N* = 66,795). The independent variable was intersectional groups, composed of 16 intersectional groups that combined various genders, races and ethnicities, and sexual orientations. The outcome variables were professional well-being (i.e., burnout, career regret) and medical school experiences (i.e., general mistreatment, discrimination, emotional climate, faculty-student interaction, faculty professionalism, and satisfaction with medical education). Given the large sample, we focused on effect sizes versus statistical significance.

**Results:**

The intersectional groups differed from each other on all professional well-being and all medical school experience variables except emotional climate, with at least small effect sizes (ηp^2^ ≥ .01). Black female sexual minority students reported the most negative outcomes on all variables. The largest differences were primarily with White male heterosexual (e.g., discrimination: *d* = 1.68, 95% CI [1.53, 1.84]) and White female heterosexual (e.g., disengagement: *d* = 0.63, 95% CI [0.48, 0.79]) students. However, being a member of a greater number of marginalized groups was not necessarily associated with more negative outcomes, and patterns of group differences varied across domains of professional well-being and medical school experiences.

**Discussion:**

Examining the combination of medical students’ gender, race and ethnicity, and sexual orientation yielded larger and more consistent effect sizes than examining each factor individually, suggesting that an intersectional approach can identify the unique challenges confronted by medical students from specific demographic groups.

## Introduction

Healthcare workers from marginalized demographic groups (e.g., racial and ethnic minorities) have reported more burnout [1] and negative work experiences such as discrimination [2] than those from dominant groups. Healthcare workers who belong to multiple marginalized groups have reported unique kinds of discrimination such as gendered racism [3,4]. Experiences of discrimination (e.g., racial discrimination) are related to greater job turnover [5], which can contribute to the underrepresentation of marginalized groups in the healthcare workforce. This may further compromise the quality of care received by patients from marginalized groups and worsen existing health disparities [6–8]. Because medical students are future physicians, it is important to study the role of demographic characteristics (e.g., gender) in their experiences. The goal of this study was to explore the roles of gender, race and ethnicity, and sexual orientation in medical students’ professional well-being and medical school experiences using an intersectional approach.

One important aspect of professional well-being is burnout, which has been defined as a syndrome caused by chronic unsuccessfully managed work stress [9]. Burnout is characterized by emotional exhaustion, depersonalization (e.g., mental distance from the job), and decreased professional efficacy [9,10]. Burnout is common in medical students, with meta-analyses indicating an average prevalence of about 40% using overall measures of burnout and 30–40% for its dimensions (e.g., emotional exhaustion, depersonalization) [11,12]. It is important to investigate medical students’ burnout because studies have shown that burnout was related to higher career regret [13] and higher dropout rates [14] among medical students, which may contribute to physician shortages and lower quality patient care [7].

Several studies have examined the relations between demographic characteristics and burnout among medical students. Results regarding gender differences in burnout among medical students have been mixed. For example, using a general measure of burnout, studies have shown that women reported more burnout [15–17] or have found no gender differences [18–20]. Results also have varied depending on the aspect of burnout being assessed. Results regarding emotional exhaustion have been mixed, with women reporting higher emotional exhaustion [16,17,21], men reporting higher emotional exhaustion [22], or no gender differences [20,23–25]. Most studies have found that men reported higher depersonalization/disengagement [17,20–22,24,25], but a few found no gender differences [16,23]. Findings have also been mixed regarding racial-ethnic differences in burnout, with results varying depending on how racial-ethnic groups were defined and which dimensions of burnout were assessed [15,16,21,24,26]. However, lesbian, gay, and bisexual (LGB) medical students consistently have reported more burnout than heterosexual students [17,21,24,27,28].

It also is important to examine the role of demographic characteristics in students’ experiences during medical school, such as discrimination and mistreatment, as well as their perceptions of the learning environment. This is important because studies have shown that around 60% of medical trainees (including medical students) reported experiencing discrimination or harassment during training [29]. Negative medical school experiences were associated with more burnout, impaired academic performance, and dropping out of medical school [14,30]. Studies consistently have suggested that students from marginalized groups (i.e., women, racial and ethnic minorities, sexual minorities) reported experiencing more mistreatment, discrimination, and microaggressions [21,31,32] and were more likely to perceive faculty as lacking respect for diversity [33]. One study [26] compared students who were and were not underrepresented in medicine (URM/URiM) in terms of the learning environment. According to the Association of American Medical Colleges [34], URM/URiM refers to students who identify as American Indian/Alaska Native, Black/African American, Hispanic/Latino/of Spanish Origin, or Native Hawaiian/Other Pacific Islander. Findings were mixed, with URiM students reporting less positive student-faculty interactions but a better emotional climate than non-URiM students [26]. One study showed that LGB students reported less favorable learning environments than did heterosexual students [27].

One recent study comprehensively examined the role of demographic characteristics in various aspects of professional well-being and school experiences among second-year and graduating (fourth-year) medical students from 2019-2022 [35]. Among the professional well-being measures (i.e., burnout, career regret), gender had a greater than small effect (ηp^2^ > 0.01) only on burnout. Specifically, women reported more exhaustion (but not disengagement) than men. The effect sizes for race and sexual orientation were less than small for both dimensions of burnout. The effects of gender, race, and sexual orientation were all less than small for career regret. For medical school experiences, both second-year and graduating gay/lesbian and bisexual students reported more discrimination than did heterosexual students. Graduating women students reported more discrimination than men, and graduating Black students reported more discrimination than did White students. However, the effects of gender, race, and sexual orientation were all less than small among both second-year and graduating students for other aspects of medical school experiences (i.e., general mistreatment, emotional climate, faculty-student interaction, faculty professionalism, satisfaction with medical education). Thus this study further demonstrated mixed findings about the role of demographic characteristics in medical students’ well-being and school experiences.

Mixed results in past research may be partly due to the common practice of studying marginalized groups in isolation, rather than using an intersectional approach (see S1 and S2 Figs in supplemental material for visual representations of these two approaches). Intersectionality is a critical theoretical framework initially used to understand Black women’s experiences of intersecting patterns of sexism and racism [36] that was later expanded to factor in the intersection among other social groups (e.g., class, sexual orientation) [37]. To be more specific, it aims to understand how interlocking systems of power (e.g., sexism, racism, heterosexism) uniquely shape the lived experiences of individuals with intersecting social positions and reinforce marginalization [36–38]. Intersectionality is an essential aspect of many theories about the experiences of people from marginalized groups, such as critical race theory and queer theory [39,40]. These theories explore how identities (e.g., race, gender, sexuality) are socially constructed and maintained and aim to understand experiences of marginalization (e.g., racism, sexism) within larger social, economic, and historical structures [39–41]. With regard to medical education, critical race theory regards racism as a structural and endemic issue in education [42]. In addition, researchers have suggested that queer identity has been pathologized in both patients and medical students/physicians, and that queer theory can be used as a framework to understand their experiences and disrupt the cis-heteronormative lens on which medical training is based [43].

It is important to explore medical students’ experiences through an intersectional lens for several reasons. First, because people are members of multiple social groups and thus vulnerable to various types of biases [40], focusing on different demographic groups in isolation can distort researchers’ understanding of the roles of demographic characteristics. Intersectional frameworks can disrupt these unidimensional conceptualizations of demographic groups, and improve understanding of social inequities [40,44]. Second, intersectional approaches can also contribute to trust and solidarity among different marginalized social groups [44]. Third, the intersectional approach has been suggested as a helpful way for medical educators to understand how various systems of power work together to affect medical students’ professional development and clinical experiences [45]. Finally, an intersectional approach can give voice to perspectives that are often ignored in medical education and scholarship [46].

We are aware of only three quantitative studies of medical students that have taken an intersectional approach and/or examined more than one identity. Two studies used data collected by the AAMC. In one study of 30,651 graduating students in 2016–2017, those from three marginalized groups (i.e., woman, non-White, LGB) reported the most burnout (in terms of exhaustion but not disengagement), mistreatment, and discrimination [21]. White, male, heterosexual students reported the lowest scores on these measures. The other study used data from graduating medical students in 2019–2021, with *n*s of ~16,000 per year [24]. This study reported burnout scores for intersectional groups that combined race-ethnicities and gender or combined racial-ethnicities and sexual orientation without conducting statistical tests of between-group differences. In a study of microaggressions among 759 medical students [31], the interaction between gender and race was not significant. However, medical students who identified as Black women reported the most microaggressions and those who identified as White men reported the fewest. Additionally, qualitative data from medical students high in stereotype threat revealed that the intersection of race with other demographic characteristics was a common theme [42]. For instance, some medical students who identified as Black and women talked about being regarded as combative, consistent with the “angry Black woman” stereotype [42].

In addition to being few in number, existing intersectional quantitative studies are limited in various respects. First, two studies only compared marginalized groups to the dominant group (e.g., White, heterosexual, male) but did not compare marginalized groups to each other [21,31]. The other study reported burnout scores for intersectional groups without significance tests [24]. None of the studies reported effect sizes. Because very small effects can be statistically significant in large samples, focusing only on significance can overestimate the roles of demographic characteristics in medical students’ experiences. Second, in papers that reported significance tests of group differences, one [21] only examined race-ethnicity as a binary variable (i.e., White vs. non-White). Racial and ethnic marginalized groups differ in their experiences [15]; grouping them together obscures these differences. Finally, only burnout, discrimination and mistreatment have been examined in intersectional studies of medical students, leaving out other important constructs, such as career regret and perceptions of the learning environment.

The aim of this study was to address these limitations by investigating the roles of gender, race and ethnicity, and sexual orientation using an intersectional approach. We also used a broad range of measures of medical students’ professional well-being (i.e., burnout and career regret) and medical school experiences (e.g., mistreatment, perception of learning environment, faculty professionalism, and satisfaction with the quality of medical education). Unlike a previous study using these data [35], the present study only used data from graduating students which provide a more complete picture of the medical school experience. In the past study [35], the effects of study year were less than small (study year was used to represent experiences from before to during the pandemic [2019–2022]). Thus, we combined years (2019–2022) to increase the sample sizes of marginalized groups in this study. We examined differences across 16 intersectional groups that combined two gender (men, women), four race-ethnicity (White, Asian, Black/African American, and Hispanic/Latino/of Spanish origin), and two sexual orientation (heterosexual, sexual minority) groups. This is a commonly-used intersectional analytic method [38] that allows researchers to compare each intersectional group with all other groups to identify the unique experiences of each group. The analyses were exploratory because intersectional research with medical students is limited and findings have been mixed in existing studies. Moreover, in other samples, some studies have shown that people from multiple marginalized groups experienced cumulative disadvantages, whereas others suggested that people from a single marginalized group (e.g., racial minority men) may experience more extreme prejudice and discrimination than those from multiple marginalized groups [47,48].

## Materials and methods

We used cross-sectional data from the 2019–2022 AAMC Graduation Questionnaire (GQ). The GQ is an annual national survey administered online to graduating (i.e., fourth year) or recently graduated students across approximately 150 US medical schools accredited by the Liaison Committee on Medical Education. Medical education in the US is traditionally a four-year graduate program, consisting of two years of preclinical science training and two years of clinical rotations [49]. Participation was voluntary with no compensation. Response rates ranged from 80% to 84% across years (total *N* = 66,795). The full sample was predominantly women (52%), White (55%), and heterosexual (91%). Regarding intersectional groups, the modal group was White, male, and heterosexual (29%; Table 1). The study was deemed exempt by the [redacted] Institutional Review Board (STUDY00019551). The Journal Article Reporting Standards for quantitative research in psychology [50] and the Strengthening the Reporting of Observational Studies in Epidemiology [51] were followed.

**Table 1:** Demographic characteristics of the sample.

Intersectional Group	Sample Size (N, Valid Percent)
Asian, Male, Heterosexual	5,631 (10%)
Asian, Male, Sexual Minority	483 (1%)
Asian, Female, Heterosexual	6,825 (13%)
Asian, Female, Sexual Minority	522 (1%)
Black, Male, Heterosexual	1,159 (2%)
Black, Male, Sexual Minority	132 (0.2%)
Black, Female, Heterosexual	2,101 (4%)
Black, Female, Sexual Minority	171 (0.3%)
Hispanic, Male, Heterosexual	1,418 (3%)
Hispanic, Male, Sexual Minority	225 (0.4%)
Hispanic, Female, Heterosexual	1,604 (3%)
Hispanic, Female, Sexual Minority	147 (0.3%)
White, Male, Heterosexual	15,709 (29%)
White, Male, Sexual Minority	1,516 (3%)
White, Female, Heterosexual	15, 296 (28%)
White, Female, Sexual Minority	1,619 (3%)
Study Year	
2019	16,656 (25%)
2020	16,630 (25%)
2021	16,609 (25%)
2022	16,900 (25%)

### Measures

#### Professional well-being.

Burnout was measured using the Oldenburg Burnout Inventory for Medical Students [52]. This measure has two eight-item subscales assessing exhaustion (e.g., “During my medical school work, I often feel emotionally drained”) and disengagement (e.g., “Lately, I tend to think less at work and do my job almost mechanically”). Items were rated on a 0 (*strongly disagree*) to 3 (*strongly agree*) scale. Total scores were calculated for each subscale (possible ranges = 0–24). Exhaustion and disengagement subscale scores have demonstrated internal consistency reliability (α = .83 and.72, respectively) and criterion validity (through relations with greater mistreatment) in a study using 2016–2018 GQ data [22]. In the current study, αs were.84 for exhaustion and.77 for disengagement.

Career regret was assessed through the question “If you could revisit your career choice, would you choose to attend medical school again” which was rated on a scale of 1 (*no*), 2 (*probably not*), 3 (*neutral*), 4 (*probably yes*), 5 (*yes*).

#### Medical school experiences.

General mistreatment and discrimination were measured by 17 items developed by AAMC about how often students reported experiencing negative behaviors from different sources (e.g., faculty, nurses) in medical school. Seven items assessed general mistreatment (e.g., “Been threatened with physical harm”) and 10 items assessed discrimination due to gender (3 items), race or ethnicity (3 items), sexual orientation (3 items), and other aspects of identity (1 item; e.g., “Been subjected to offensive remarks/names related to sexual orientation”). Items were rated on 1 *(Never*) to 4 (*Frequently*) scale. Total scores were calculated with possible ranges of 7–28 for mistreatment and 10–40 for discrimination. Scores on both measures have demonstrated criterion validity through relations with higher exhaustion and disengagement in a study that analyzed 2016 and 2017 AAMC GQ data [21]. In the current study, αs were.59 for general mistreatment and.77 for discrimination.

The learning environment was assessed using the Medical Student Learning Environment Survey [53]. This measure has two dimensions: emotional climate (3 items; e.g., “The educational experience makes students feel a sense of achievement”) and student-faculty interactions (4 items; e.g., “Faculty are helpful to students seeking advice not directly related to academic matters”). Items are rated on a 0 (*Never*) to 5 (*Always*) scale. The total score range was 0–15 for emotional climate and 0–20 for student-faculty interactions. Scores on the emotional climate (α = .92) and student-faculty interactions (α = .79) subscales demonstrated internal consistency reliability in a study using AAMC data from second-year medical students [22]. Subscale scores have also demonstrated criterion validity, with less favorable emotional climate and faculty-student interactions related to higher burnout in a study using 2016–2017 AAMC GQ data [26]. In the current study, αs were.94 for emotional climate and.80 for student-faculty interactions.

Faculty professionalism was assessed by 11 items developed by AAMC regarding how often medical school faculty demonstrated professional attitudes or behaviors (e.g., “Being respectful of others”). Items were rated on 1 (*Never*) to 6 (*Always*) scale. Total scores were calculated (range = 11–66, α = .87).

Satisfaction with medical education was assessed by one question (“Overall, I am satisfied with the quality of my medical education”) which was rated on a 1 (*Strongly disagree*) to 5 (*Strongly agree*) scale.

#### Predictor variable.

We created the intersectional group variable based on self-reported race and ethnicity, gender, and sexual orientation. For race and ethnicity, response options included American Indian or Alaska Native, Asian, Black or African American, Hispanic/Latino/of Spanish origin, Native Hawaiian or Other Pacific Islander, White, and Other. Those who identified solely as American Indian/Alaska Native or as Native Hawaiian/Other Pacific Islander were excluded from analyses due to small sample sizes (i.e., 0.1% of the sample). The intersectional groups were even smaller (e.g., only two participants identified as Native Hawaiian/Other Pacific Islander, sexual minority, and men). Students who selected more than one racial and ethnic group and who selected “Other” were also excluded due to ambiguity about their race/ethnicity. Information on gender was from students’ AAMC accounts, where they had the option of endorsing female/woman, male/man, or decline to answer, which confounded gender and sex. Those who selected “decline to answer” were excluded due to the small sample size (0.03% of the sample). The research team contacted the AAMC for gender identity data but the data were not available owing to privacy concerns of gender minority students. For sexual orientation, the response options were heterosexual or straight, gay or lesbian, and bisexual in the 2019–2021 GQ surveys. These choices were expanded to include asexual, pansexual, queer, and other options in the 2022 GQ. Among students who did not identify as heterosexual, the largest group was bisexual, which was 5% of the total sample. The sample sizes of intersectional groups were even smaller (e.g., only one person identified as a Black, pansexual, man). To ensure adequate sample sizes, and to avoid identifying individuals from very small groups, those who identified as gay or lesbian, bisexual, asexual, pansexual, queer, and other were recoded as “sexual minority.” In sum, the intersectional group variable had 16 categories, with all combinations of the four-category race and ethnicity (White, Asian, Black, Hispanic), binary gender (man, woman), and binary sexual orientation (heterosexual, sexual minority) variables (e.g., Asian heterosexual woman).

### Analysis plan

We conducted all analyses using SPSS Version 27. Scores greater than three standard deviations (SDs) above or below the mean were considered outliers and winsorized to the nearest existing scores within 3 SDs of the mean. Listwise deletion was used to address missing data given the large sample size, the small amount of missing data, and minimal differences between those with and without complete data (see S1 and S2 Tables). For professional well-being, we performed three univariate analyses of covariance (ANCOVA) with exhaustion, disengagement, and career regret as outcome variables. For medical school experiences, we performed six ANCOVAs with emotional climate, student-faculty interactions, general mistreatment, discrimination, faculty professionalism, and satisfaction with medical education as outcomes. The predictor was the intersectional group variable. Because gender, race and ethnicity, and sexual orientation differed across study years with greater than small effect sizes (Phi and Cramer’s V > .10) [54], study year (reflecting time periods before and during the COVID 19 pandemic) was included as a covariate. Given the large sample size, we focused on effect sizes rather than statistical significance, and used the following conventions to evaluate ηp^2^ effect sizes for the ANCOVAs: small (0.01), medium (0.06), and large (0.14) [54]. If univariate effects were at least small, we calculated Cohen’s *d* effect sizes to compare group means using the following conventions: small (0.20), medium (0.50), and large (0.80) [54]. Only effect sizes that were at least small were regarded as meaningful. Less than small effect sizes were regarded as indicating no (meaningful) between-group differences. Because the study focused on effect sizes instead of statistical significance, *p* values were not corrected to account for multiple comparisons. The SPSS syntax is available in OSF: https://tinyurl.com/328vtzdz.

## Results

All outcome variables were normally distributed (i.e., absolute skewness < 2 or absolute kurtosis < 7) [55]. The ANCOVA assumption of homogeneity of regression slopes was met for all outcome variables. Although the assumption of homogeneity of variance was not met, perhaps due to the very large sample size, ANCOVAs are relatively robust to violations of this assumption when the largest sample variance is no more than three times the smallest sample variance, which was true for all outcome variables [56]. Mean scores and SDs across the 16 intersectional groups for all outcome variables are in Tables 2 and 3.

**Table 2:** Unadjusted means and standard deviations (SD) for each intersectional group for professional well-being.

	Exhaustion		Disengagement		Career Regret	
	Mean	SD	Mean	SD	Mean	SD
Asian, Male, Heterosexual	11.17	3.75	10.56	3.79	4.09	0.99
Asian, Male, Sexual Minority	12.15	3.84	11.24	3.79	3.88	1.03
Asian, Female, Heterosexual	11.89	3.67	9.95	3.58	4.17	0.94
Asian, Female, Sexual Minority	12.68	3.40	10.66	3.54	4.01	0.97
Black, Male, Heterosexual	10.82	3.52	10.03	3.49	4.22	0.95
Black, Male, Sexual Minority	11.65	3.88	10.74	3.76	4.02	1.00
Black, Female, Heterosexual	12.39	3.62	10.19	3.55	4.06	1.03
Black, Female, Sexual Minority	13.04	3.78	11.44	4.09	3.84	1.08
Hispanic, Male, Heterosexual	10.87	3.93	9.69	3.76	4.24	0.96
Hispanic, Male, Sexual Minority	11.91	3.94	10.96	3.80	4.11	0.97
Hispanic, Female, Heterosexual	12.02	3.67	9.26	3.49	4.24	0.93
Hispanic, Female, Sexual Minority	12.53	3.76	9.58	3.52	4.16	0.96
White, Male, Heterosexual	10.26	3.77	9.91	3.80	4.28	0.91
White, Male, Sexual Minority	11.41	3.79	10.57	3.69	4.15	0.94
White, Female, Heterosexual	11.05	3.70	9.13	3.65	4.35	0.87
White, Female, Sexual Minority	11.92	3.78	9.85	3.87	4.27	0.92

**Table 3:** Unadjusted means and standard deviations (SD) for each intersectional group for medical school experiences.

	General Mistreatment		Discrimination		Emotional Climate		Faculty- Student Interaction		Faculty Professionalism		Satisfaction with Medical Education	
	Mean	SD	Mean	SD	Mean	SD	Mean	SD	Mean	SD	Mean	SD
Asian, Male, Heterosexual	7.88	1.45	10.61	1.52	9.90	3.40	14.25	3.47	53.69	7.23	4.16	0.79
Asian, Male, Sexual Minority	8.21	1.69	11.10	2.05	9.29	3.30	13.93	3.62	51.87	7.41	4.07	0.82
Asian, Female, Heterosexual	8.05	1.50	11.02	1.83	9.56	3.15	14.04	3.33	52.59	6.58	4.16	0.73
Asian, Female, Sexual Minority	8.50	1.75	11.75	2.41	8.52	3.17	13.25	3.35	50.72	6.76	4.07	0.77
Black, Male, Heterosexual	7.92	1.45	10.85	1.72	10.10	3.32	14.20	3.42	53.15	7.15	4.23	0.78
Black, Male, Sexual Minority	8.16	1.48	11.27	2.10	9.35	3.01	14.17	3.41	51.34	6.93	4.23	0.71
Black, Female, Heterosexual	8.34	1.65	11.51	2.22	9.16	3.28	13.53	3.46	51.06	6.71	4.06	0.78
Black, Female, Sexual Minority	8.83	1.90	12.40	2.60	8.44	3.45	13.17	3.80	49.53	7.09	3.97	0.81
Hispanic, Male, Heterosexual	7.88	1.39	10.60	1.45	10.12	3.45	14.37	3.64	54.30	6.95	4.23	0.82
Hispanic, Male, Sexual Minority	8.22	1.60	11.37	2.13	9.04	3.69	13.64	3.80	51.80	7.00	4.08	0.90
Hispanic, Female, Heterosexual	8.17	1.56	11.05	1.82	9.82	3.31	14.07	3.58	53.40	6.60	4.23	0.73
Hispanic, Female, Sexual Minority	8.47	1.80	11.77	2.43	8.64	3.53	13.27	3.75	51.61	6.65	3.97	0.87
White, Male, Heterosexual	7.87	1.39	10.38	1.18	9.81	3.26	14.78	3.23	53.82	6.20	4.29	0.76
White, Male, Sexual Minority	8.31	1.61	10.82	1.65	9.18	3.21	14.39	3.33	52.30	6.15	4.23	0.79
White, Female, Heterosexual	8.15	1.49	10.76	1.40	9.64	3.10	14.75	3.17	52.84	5.75	4.33	0.70
White, Female, Sexual Minority	8.56	1.68	11.49	2.02	8.69	3.16	14.13	3.31	51.00	6.18	4.22	0.74

**Fig 1:**
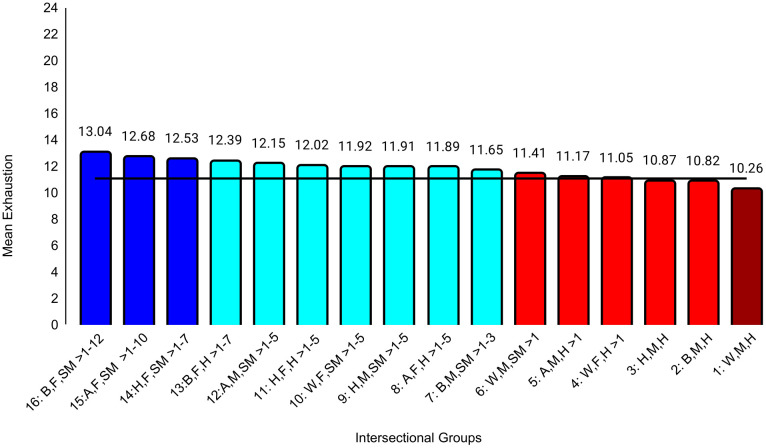
Intersectional group differences in exhaustion. Color: Blue = 3 marginalized groups, cyan = 2 marginalized groups, red = 1 marginalized group, maroon = no marginalized groups. Race coding: B = Black, A = Asian, H = Hispanic, W = White; Gender coding: F = Female, M = Male; Sexual orientation coding: SM = Sexual Minority, H = Heterosexual. Each intersectional group is numbered and intersectional groups indicated as> differ by at least Cohen’s *d* = 0.20. Black line = observed grand mean.

**Fig 2:**
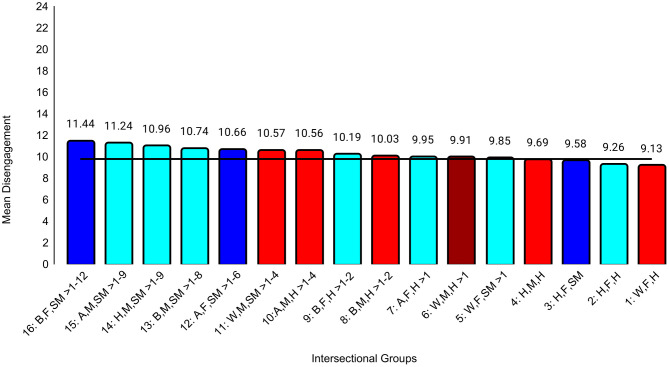
Intersectional group differences in disengagement. Color: Blue = 3 marginalized groups, cyan = 2 marginalized groups, red = 1 marginalized group, maroon = no marginalized group. Race coding: B = Black, A = Asian, H = Hispanic, W = White; Gender coding: F = Female, M = Male; Sexual orientation coding: SM = Sexual Minority, H = Heterosexual. Each intersectional group is numbered and intersectional groups indicated as> differ by at least Cohen’s *d* = 0.20. Black line = observed grand mean.

**Fig 3:**
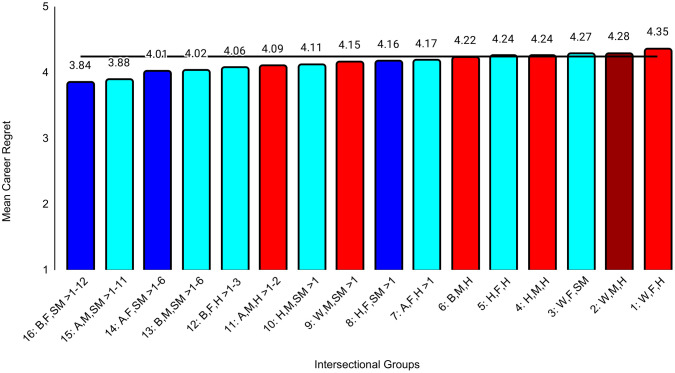
Intersectional group differences in career regret. Color: Blue = 3 marginalized groups, cyan = 2 marginalized groups, red = 1 marginalized group, maroon = no marginalized group. Race coding: B = Black, A = Asian, H = Hispanic, W = White; Gender coding: F = Female, M = Male; Sexual orientation coding: SM = Sexual Minority, H = Heterosexual. Each intersectional group is numbered and intersectional groups indicated as> differ by at least Cohen’s *d* = 0.20. Black line = observed grand mean.

The first set of ANCOVAs assessed differences across intersectional groups in professional well-being (Table 4). The effect sizes were at least small for all three outcomes: exhaustion (ηp^2^ = .031), disengagement (ηp^2^ = .019), and career regret (ηp^2^ = .012). The effect sizes for all between-group differences are in S3 Table. For example, the biggest group difference for exhaustion was between Black female sexual minority (highest exhaustion) and White male heterosexual (*d* = 0.74, 95% CI [0.58, 0.89]) students, who reported the lowest exhaustion. The largest differences for disengagement (*d* = 0.63, 95% CI [0.48, 0.79]) and career regret (*d* = 0.58, 95% CI [0. 43, 0.73]) were between Black female sexual minority (highest disengagement, career regret) and White female heterosexual students (lowest disengagement, career regret). These represent moderate to large differences. All effects of study year were less than small (Table 4). Between-group unadjusted differences that were at least small are specified in Figs 1–3.

**Table 4:** Univariate tests for professional well-being and medical school experiences.

	Exhaustion (*N* = 53,758)				Disengagement (*N* = 53,762)				Career Regret (*N* = 54,512)			
	F (df)		ηp2		F (df)		ηp2		F (df)		ηp2	
**Professional Well-being**												
Study year	0.26 (1)		.000		8.93 (1)		.000		103.82 (1)		.002	
Intersectional group	116.19 (15)		**.031**		70.14 (15)		**.019**		43.71 (15)		**.012**	
	General Mistreatment (*N* = 54,202)		Discrimination (*N* = 53,863)		Emotional Climate (*N* = 54,271)		Faculty-Student Interaction (*N* = 54,002)		Faculty Professionalism (*N* = 53,724)		Satisfaction (*N* = 52,952)	
	F (df)	ηp2	F (df)	ηp2	F (df)	ηp2	F (df)	ηp2	F (df)	ηp2	F (df)	ηp2
**Medical School Experiences**												
Study year	70.56 (1)	.001	.08 (1)	.000	.01 (1)	.000	18.70 (1)	.000	11.14 (1)	.000	9.40 (1)	.000
Intersectional group	56.87 (15)	**.016**	176.50 (15)	**.047**	31.57 (15)	.009	48.80 (15)	**.013**	63.78 (15)	**.018**	44.03 (15)	**.012**

*Note.* Bold = ηp2 greater than.01. Adjusted R squared for exhaustion = .031, for disengagement = .019, for career regret = .014, for general mistreatment = .016, for discrimination = .047, for emotional climate = .008, for faculty-student interaction = .014, for faculty professionalism = .017, and for satisfaction = .012.

**Fig 4:**
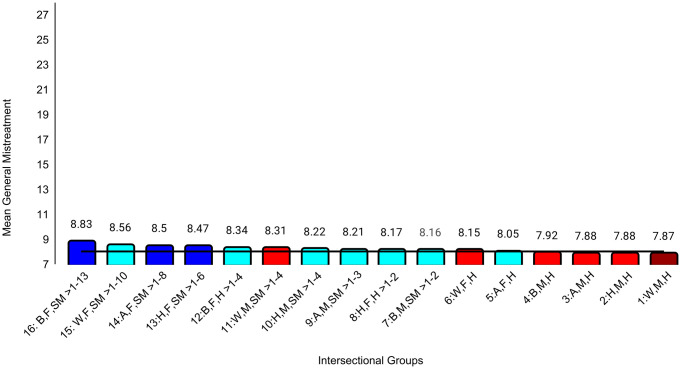
Intersectional group differences in general mistreatment. Color: Blue = 3 marginalized groups, cyan = 2 marginalized groups, red = 1 marginalized group, maroon = no marginalized group. Race coding: B = Black, A = Asian, H = Hispanic, W = White; Gender coding: F = Female, M = Male; Sexual orientation coding: SM = Sexual Minority, H = Heterosexual. Each intersectional group is numbered and intersectional groups indicated as> differ by at least Cohen’s *d* = 0.20. Black line = observed grand mean.

**Fig 5:**
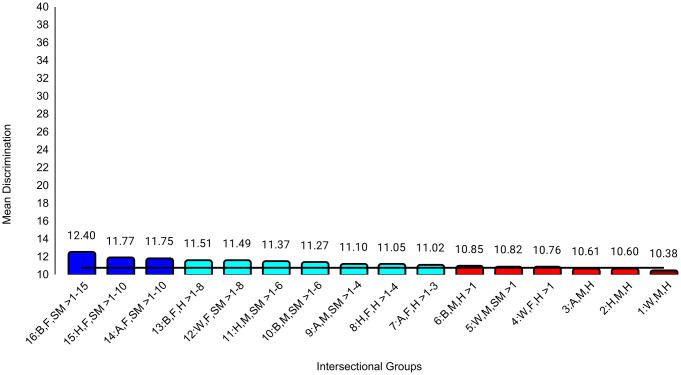
Intersectional group differences in discrimination. Color: Blue = 3 marginalized groups, cyan = 2 marginalized groups, red = 1 marginalized group, maroon = no marginalized group. Race coding: B = Black, A = Asian, H = Hispanic, W = White; Gender coding: F = Female, M = Male; Sexual orientation coding: SM = Sexual Minority, H = Heterosexual. Each intersectional group is numbered and intersectional groups indicated as> differ by at least Cohen’s *d* = 0.20. Black line = observed grand mean.

**Fig 6:**
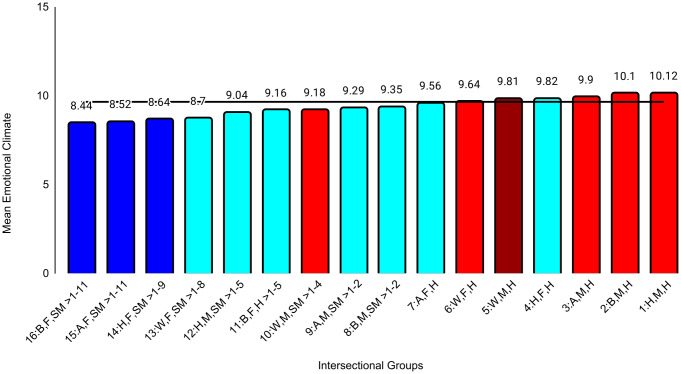
Intersectional group differences in emotional climate. Color: Blue = 3 marginalized groups, cyan = 2 marginalized groups, red = 1 marginalized group, maroon = no marginalized group. Race coding: B = Black, A = Asian, H = Hispanic, W = White; Gender coding: F = Female, M = Male; Sexual orientation coding: SM = Sexual Minority, H = Heterosexual. Each intersectional group is numbered and intersectional groups indicated as> differ by at least Cohen’s *d* = 0.20. Black line = observed grand mean.

**Fig 7:**
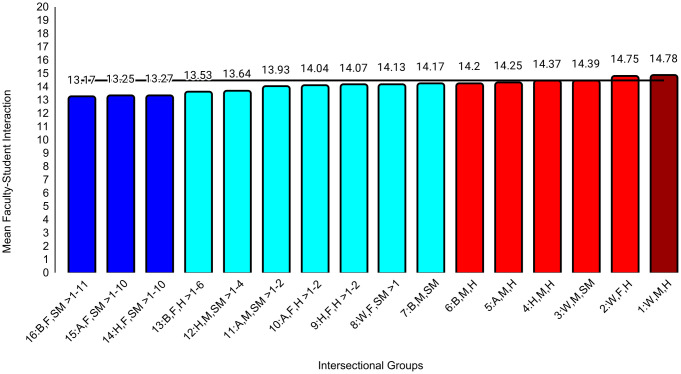
Intersectional group differences in faculty-student interaction. Color: Blue = 3 marginalized groups, cyan = 2 marginalized groups, red = 1 marginalized group, maroon = no marginalized group. Race coding: B = Black, A = Asian, H = Hispanic, W = White; Gender coding: F = Female, M = Male; Sexual orientation coding: SM = Sexual Minority, H = Heterosexual. Each intersectional group is numbered and intersectional groups indicated as> differ by at least Cohen’s *d* = 0.20. Black line = observed grand mean.

**Fig 8:**
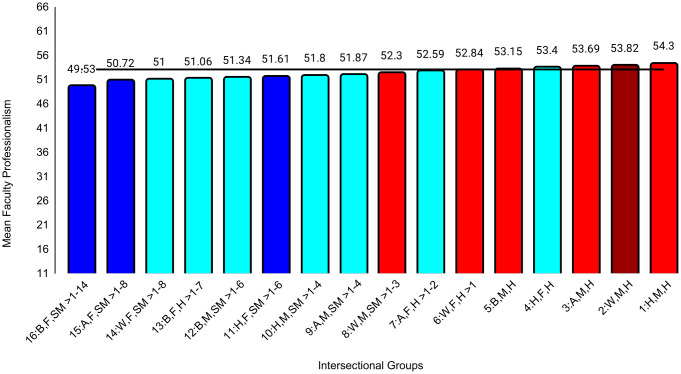
Intersectional group differences in faculty professionalism. Color: Blue = 3 marginalized groups, cyan = 2 marginalized groups, red = 1 marginalized group, maroon = no marginalized group. Race coding: B = Black, A = Asian, H = Hispanic, W = White; Gender coding: F = Female, M = Male; Sexual orientation coding: SM = Sexual Minority, H = Heterosexual. Each intersectional group is numbered and intersectional groups indicated as> differ by at least Cohen’s *d* = 0.20. Black line = observed grand mean.

**Fig 9:**
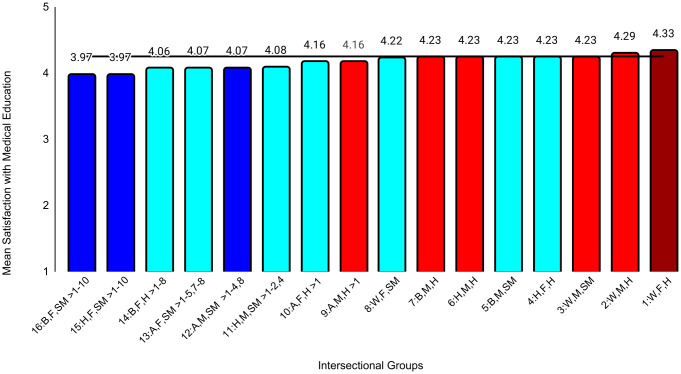
Intersectional group differences in satisfaction with medical education. Color: Blue = 3 marginalized groups, cyan = 2 marginalized groups, red = 1 marginalized group, maroon = no marginalized group. Race coding: B = Black, A = Asian, H = Hispanic, W = White; Gender coding: F = Female, M = Male; Sexual orientation coding: SM = Sexual Minority, H = Heterosexual. Each intersectional group is numbered and intersectional groups indicated as> differ by at least Cohen’s *d* = 0.20. Black line = observed grand mean.

In the second set of ANCOVAs, the intersectional group variable had at least small effects for all medical school experience variables except emotional climate (ηp^2 ^= .009). The largest effect was for discrimination (ηp^2^ = .047). Effect sizes for all between-group differences are in S4 Table. Examining means for each outcome, Black female sexual minority students reported the most negative experiences in all categories. The largest group differences for general mistreatment (*d* = 0.69, 95% CI [0.54, 0.84]), discrimination (*d* = 1.68, 95% CI [1.53, 1.84], and perceptions of faculty-student interactions (*d* = 0.50, 95% CI [0.34, 0.65]) were with White male heterosexual students, who reported the most positive experiences on these three variables. The largest differences for perceptions of faculty professionalism (*d* = 0.69, 95% CI [0.52, 0.85]) were with Hispanic male heterosexual students, who reported the most positive experiences. The largest differences for satisfaction with education (*d* = 0.52, 95% CI [0.37, 0.67]) were with White female heterosexual students, who were most satisfied. As with professional well-being, all these group differences were moderate to large. All effects of study year were less than small (Table 4). Between-group differences that were at least small are specified in Figs 4–9.

## Discussion

The primary goal of this exploratory study was to examine US medical students’ experiences using an intersectional approach. Results suggested that examining the combination of medical students’ gender, race and ethnicity, and sexual orientation yielded larger and more consistent between-group differences for professional well-being and medical school experiences than when each factor was examined individually using the same dataset [35]. Although the majority of effects associated with the individual variables were less than small in the previous study [35], the intersectional analyses reported here revealed specific groups that reported much worse experiences than others. Specifically, Black female sexual minority students reported the most negative outcomes on all variables. They differed from most other groups with at least small effect sizes, sometimes even from other multiple marginalized groups (e.g., Asian and Hispanic female sexual minorities), and they reported more discrimination than *all* other groups. This is consistent with past research showing that Black women medical students experienced more mistreatment than their URM peers [57]. Additionally, there were minimal differences in medical students’ professional well-being and school experiences as a function of study year (i.e., before vs. during the pandemic). When examining gender, race and ethnicity, and sexual orientation intersectionally, it is even more evident that the role of demographic groups in medical students’ experiences was greater than that of the COVID-19 pandemic, as found in past research in which demographic groups were examined separately [35].

Another goal was to examine a wider range of outcomes than in other intersectional studies [21,24,31]. Our exploratory analyses revealed different patterns of group differences across various domains of professional well-being and medical school experiences. On the burnout subscales, there was a clear pattern in which belonging to more marginalized groups was associated with higher exhaustion, but there was no clear pattern for disengagement. For example, one group with three marginalized characteristics (Hispanic female sexual minority students) reported relatively low levels of disengagement that did not differ from those from no apparent marginalized groups (White male heterosexual students). Research that has not taken an intersectional approach generally has found that female students reported lower levels of disengagement than male students [17,20,21,25]. However, our intersectional analyses showed differences between female groups that differed in terms of race and ethnicity and/or sexual orientation, underscoring the importance of this approach. For medical school experiences, there was a clear pattern in which belonging to more marginalized groups was associated with more discrimination, perhaps because the discrimination questions focused on gender, race, and sexual orientation. This pattern was less clear for mistreatment, although Black female sexual minority students reported the most mistreatment and White male heterosexual students reported the least. However, levels of mistreatment and discrimination were generally low. Faculty-student interactions were another aspect of medical school experiences that showed a clear pattern in which belonging to more marginalized groups was associated with worse perceptions of those interactions. This is consistent with previous studies in which URM students reported less favorable faculty-student interactions than did White students [26] and LGB students reported less favorable faculty-student interactions than did heterosexual students [27].

One reason that belonging to a higher number of marginalized groups was not necessarily related to more negative outcomes may relate to *intersectional invisibility*. Researchers have proposed that, when people belong to more than one marginalized group, they may be more “invisible” than those from a single marginalized group because people from multiple marginalized groups do not necessarily fit the prototypes of the constituent marginalized groups (e.g., Asian woman vs. Asian or woman) [48]. This invisibility has both advantages and disadvantages and their combination may make it more difficult to predict what roles different demographic characteristics will play [48]. Because this study was exploratory, we did not have a priori hypotheses about whether we would see different patterns of group differences for different aspects of professional well-being and medical school experiences. Future research should attempt to replicate our findings and explore potential explanations for different patterns across outcome measures.

Our findings also illustrate the need to separately analyze racial and ethnic marginalized groups. An intersectional study that categorized students only as White or non-White [21] showed that any groups that included women reported significantly lower disengagement (less burnout) than did White male heterosexual students. However, in our study, only White female heterosexual students reported lower disengagement than White male heterosexual students, and Black female sexual minority students reported *higher* disengagement. Relatedly, although Black and Hispanic students are both URM and usually grouped together [26], their experiences differed. For example, Black female sexual minority students reported more disengagement, career regret, mistreatment, and discrimination and lower faculty professionalism than did Hispanic female sexual minority students.

Although Asian medical students are not considered URM/URiM according to the AAMC [34], they also encountered many challenges, consistent with prior research [58]. For instance, Asian female sexual minority students’ professional well-being and medical school experiences generally were as negative as those of Black female sexual minorities. The differences between Asian and Hispanic female sexual minority students were not meaningful except for disengagement, with Asian female sexual minority students reporting *more* disengagement. Thus, although Asian students are not URM, their experiences can be as challenging, especially for those from other marginalized groups.

Our findings have several implications for US medical schools. Using an intersectional approach highlights the need for research on the unique challenges and experiences faced by US medical students from specific multiply-marginalized groups [57]. Relatedly, our findings suggest that US medical school administrators should consider tailoring system-level interventions to students from different intersectional groups. One system-level intervention is to recruit faculty from multiply-marginalized groups (e.g., Black women) who can serve as role models for medical students with similar backgrounds and promote interactions between faculty and students [59]. Another potential system-level intervention is to implement intersectional mentorship. This includes recognizing how mentors’ and mentees’ intersectional social identities shape their experiences and relationships, and helping mentees navigate experiences of oppression [60]. Importantly, intersectional mentorship should move beyond the mentor-mentee dyad to also address inequities in academic institutions and academic culture [45,60]. This might involve advocating for mentees and including mentees in decision-making processes such as search committees [60]. Our findings can inform intersectional mentorship efforts by helping mentors better understand the unique experiences and needs of various intersectional groups and which groups may be at risk of more difficult medical school experiences in the US medical school context. Although the specific groups at particular risk may differ across countries, the need to address intersectionality in medical education is a global issue [61] as countries across the globe are perceived as becoming increasingly diverse [62].

Several study limitations also should be noted. First, the gender data were students’ responses to a list of options including “female”, “male”, and “decline to answer.” This question confounded gender and sex, and participants who chose “decline to answer” were excluded because of the small sample size. Because gender and sex can play different roles in health (including mental health), conflating them can make it difficult to interpret results. These responses also fail to recognize the experiences of nonbinary individuals [63].

Second, there were limitations with regard to the analysis of race and ethnicity. For example, given small sample sizes, we excluded two groups (Native Hawaiian/other Pacific Islander; American Indian/Alaska Native). Considering the large overall sample size and high response rates, these small sample sizes reflect the extent to which these two groups have been historically excluded from medical school [64–66]. The decision to remove these groups from analyses may further exacerbate the exclusion and marginalization experienced by these racial and ethnic groups. Additionally, students (11% of sample) who selected more than one racial/ethnic group and who selected “Other” were removed because of ambiguity about their race/ethnicity and difficulties in interpreting results. Excluding them compromised the representativeness of the sample and the generalizability of the results. Finally, the racial and ethnic groups we included (e.g., Asian, Hispanic) were treated as monolithic groups, despite their varied representativeness. For instance, 33% of Asian medical students in the US identified as Indian yet only 2% identified as Bangladeshi [67]. Among US medical school applicants who identified as Hispanic, Latino, or of Spanish origin, 28.1% identified as Mexican, Mexican American, or Chicano/Chicana, and only 1.2% identified as Argentinean [68]. Grouping them together may overlook the experiences of specific underrepresented racial-ethnic groups.

Third, we dichotomized sexual minority status because of the small sample sizes of different sexual minority groups, especially when intersectional groups were created. However, sexual minority groups differ greatly from each other in many aspects, such as rates of sexual identity disclosure [69]. Additionally, bisexual students have reported less favorable perceptions of the learning environment than gay or lesbian students [27]. Consequently, grouping all sexual minority groups together may obscure important differences among them.

Fourth, only gender, race and ethnicity, and sexual orientation were examined; however, other demographic characteristics, such as socioeconomic and disability status [30], may also play a role in medical students’ experiences. For instance, in one study, Asian and URM students with disabilities were at greater risk of burnout than were White students with disabilities [70]. Another study that examined the intersectional roles of disability status, family income, and race and ethnicity found that medical students who identified as non-White, low-income, and with disabilities were more likely to take a leave of absence in medical school compared with other intersectional groups [71]. Future research should take these additional factors into account for a more comprehensive examination of intersectionality.

There also are limitations associated with the study methods. For example, the items in the general mistreatment measure were heterogeneous (e.g., threatened with physical harm, subjected to unwanted sexual advances) and the internal consistency of the measure was relatively low. As a result, examining the items altogether may not accurately reflect the role of demographic characteristics in medical students’ experiences of mistreatment. In addition, the cross-sectional nature of the study has limitations, including that we cannot draw causal conclusions. Focusing on demographic group differences also does not reveal the systemic factors underlying those differences. Moreover, using data from graduating students excludes those who dropped out, and it may be difficult for students to recall experiences over a four-year time frame. Finally, for intersectional studies, a mixed-methods approach that combines quantitative analyses and culturally-grounded narratives may promote better understanding of the experiences of marginalized groups [72].

In conclusion, ours is the largest intersectional study of US medical students’ experiences to date and highlights the importance of this approach. Using an intersectional lens might be useful for US medical school administrators when considering the experiences of medical students and developing interventions to improve the experiences of all medical students.

## Supporting information

S1 Table Missing values in the sample data. (XLSX)

S2 Table Comparing professional well-being and medical school experiences of participants with and without intersectional identity variable. (XLSX)

S3 Table Effect sizes for professional well-being. (XLSX)

S4 Table Effect sizes for medical school experiences. (XLSX)

S1 Fig Studying the role of gender, race, and sexual orientation separately. (TIF)

S2 Fig Studying the role of gender, race, and sexual orientation intersectionally. (TIF)
