# Supplementary figures and images for: The role of demographic characteristics in US medical students’ professional well-being and medical school experiences: An intersectional approach
Source: PLoS One. 2025 Dec 16;20(12):e0338906. doi: 10.1371/journal.pone.0338906 (PMC12707665; doi:10.1371/journal.pone.0338906)

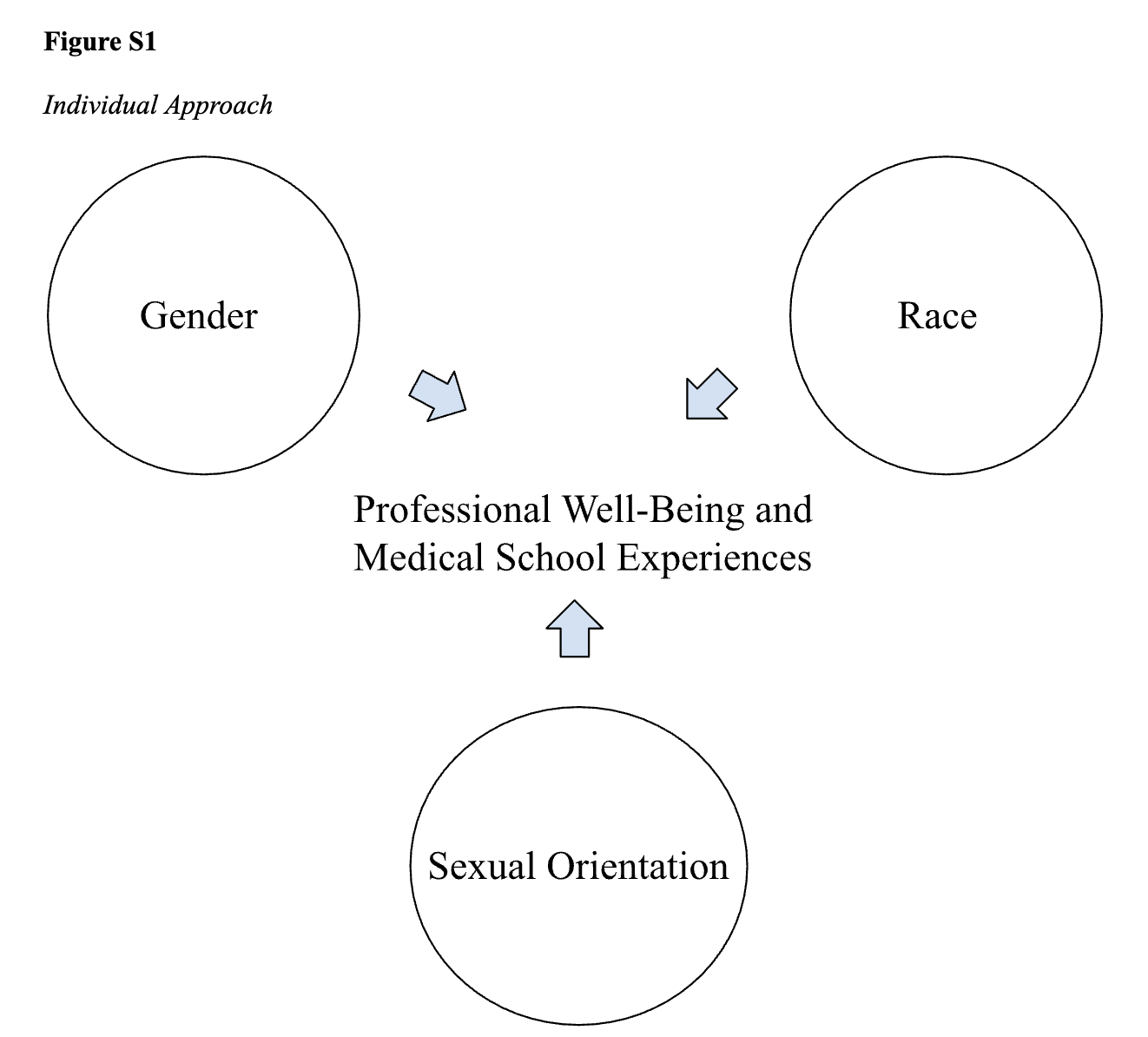

Supplement: S1 Fig — (TIF) [file pone.0338906.s005.tif]

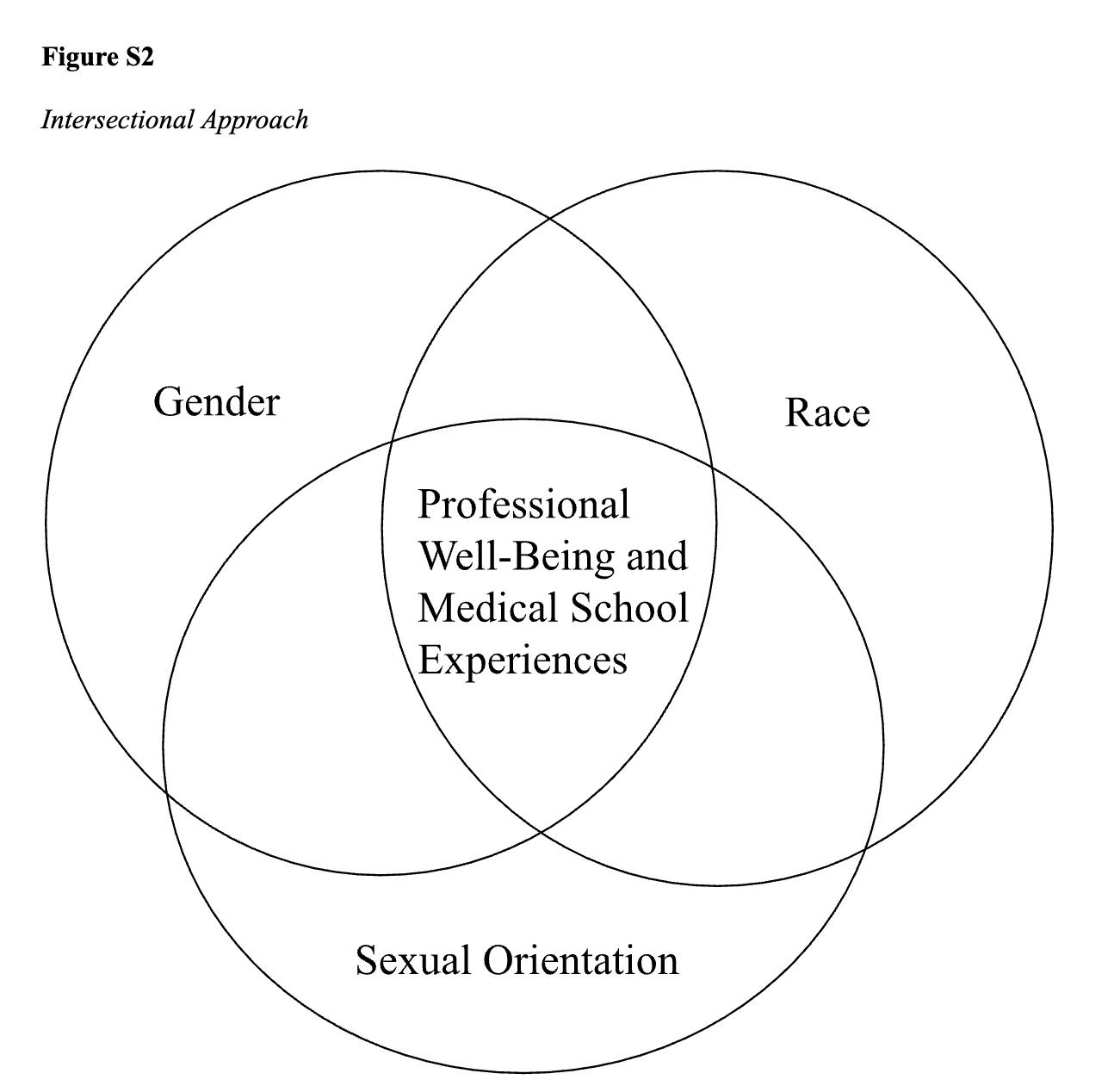

Supplement: S2 Fig — (TIF) [file pone.0338906.s006.tif]
